# Perceptions of professional challenges by emergency medical services providers: a qualitative content analysis study

**DOI:** 10.1186/s12873-024-00955-6

**Published:** 2024-03-06

**Authors:** Afshin Khazaei, Ali Afshari, Mahnaz Khatiban, Seyed Reza Borzou, Khodayar Oshvandi, Majedeh Nabavian, Maryam Maddineshat

**Affiliations:** 1 Department of Medical Emergencies, Asadabad School of Medical Sciences, Asadabad, Iran; 2 Chronic Diseases (Home Care) Research Center, Department of Medical Surgical Nursing, School of Nursing and Midwifery, Hamadan University of Medical Sciences, Hamadan, Iran; 3 Mother and Child Care Research Center, Department of Medical Surgical Nursing, School of Nursing and Midwifery, Hamadan University of Medical Sciences, Hamadan, Iran; 4 Mother and Child Care Research Center, Department of Nursing, Hamadan University of Medical Sciences, Hamadan, Iran; 5 Department of Nursing and Midwifery, Comprehensive Health Research Center, Babol Branch, Islamic Azad University, Babol, Iran; 6 Department of Nursing, School of Malayer Nursing, Chronic Disease (Home Care) Research Center, Hamadan University of Medical Sciences, Hamadan, Iran

**Keywords:** Emergency medical services providers, Professional challenge, Pre-hospital field

## Abstract

**Introduction:**

Emergency medical services (EMS) providers encounter a variety of challenges due to the unpredictable, uncontrollable, and dynamic conditions in the pre-hospital field. This study explored the perceived professional challenges among EMS providers.

**Materials and methods:**

This study was conducted using a qualitative research approach and the method of content analysis. Eighteen EMS providers were purposively selected from EMS stations in Hamadan, Iran. The collected data were then analyzed based on the Granheim and Lundman's method.

**Results:**

Based on data analysis, five categories and one theme were identified. The extracted theme was professional challenges. The five categories were as follows: Ineffective policies; multicultural and multidisciplinary factors; ambulance dispatch route problems; legal issues; and abuse against the emergency medical services

**Conclusion:**

In general, it has been found that EMS providers encounter numerous and complex professional challenges during their work. EMS managers can utilize the findings of the present study to develop strategies for reducing the professional challenges faced by EMS providers. By doing so, they can improve the quality of care in the prehospital field.

## Introduction

The emergency medical services (EMS) plays a vital role in the health care system [1]. Providing care to patients in the pre-hospital field is different from the hospital environment for reasons such as being in unpredictable situations, lack of personnel and hospital equipment resources, not having complete information from patients, providing care to patients in their own living place, providing care in public communities, and care at crime scenes [2, 3]. All health care providers face challenges when providing care to patients, and in the pre-hospital field, emergency medical services (EMS) providers are no exception to this rule [4]. Pre-hospital care presents unique complexities compared to hospital care, and EMS providers in this field are more likely to encounter additional challenges compared to hospital staffs [5].

Emergency medical service providers in Iran are also at the forefront of the healthcare system and operate under the Medical Emergency Organization, providing emergency care to patients and accident victims. Multiple studies have been conducted on pre-hospital care in Iran, with some indicating that emergency medical service providers experience high levels of job stress [6, 7]. Other studies have shown that musculoskeletal injuries are more common in this group than in hospital nurses [8]. One study identified crisis conditions and personal and professional conflicts as the main sources of work-related stress for emergency medical service providers [9]. Another study identified health-threatening factors during missions as a major source of stress in this profession [10]. A study on ethical decision-making in pre-hospital care found that barriers included understanding the situation, patient-related factors, input-output imbalance, and inconsistencies in the healthcare system [11]. While most studies in Iran have been quantitative, there has been a lack of qualitative research on identifying professional challenges faced by emergency medical service providers.

Identifying these challenges requires conducting qualitative studies. Qualitative studies can help achieve a deep understanding and identify various aspects of a concept, as qualitative approaches examine a concept in its cultural context from the perspectives of individuals who have had long engagement with the concept in question [12]. Therefore, a qualitative approach can be used to discover and better understand the professional challenges in the work environment of medical emergency personnel. On the other hand, improving and enhancing the quality of care in pre-hospital field will not be possible without understanding the professional challenges through the experiences of EMS providers. Accordingly, the present study was conducted using a qualitative approach to provide a deep description of the challenges facing EMS providers in one of the cities located in western Iran.

## Materials and methods

### Study design and setting

This qualitative study was conducted using the conventional content analysis approach. Content analysis is an analytical approach that provides insights into understanding the phenomenon being studied [13]. We have followed the COREQ guidelines when reporting the findings of this qualitative study [14]. The present study was conducted in the emergency medical system of Hamadan, a western city in Iran. The Emergency Medical Services in Iran were established in 1978. This organization is a part of the Ministry of Health, Treatment, and Medical Education. EMS delivery is funded by the government and is provided free of charge. At the beginning of the establishment of Emergency Medical Services, staff members would start working after completing a six-month theoretical and practical course. In 2002, some universities of medical sciences began to student admission in the field of emergency medicine, and in 2006, the admission of emergency medicine students in universities of medical sciences across the country began in two levels of "associate" and "bachelor" only for male candidates. In 2020, admission conditions for pre-hospital emergency medicine were announced for both male and female candidates. Participants are invited to take part in the interview stages, examination, psychological tests, and physical readiness after passing the theoretical exam. Some special admission conditions in this field include: not suffering from chronic, infectious, or incurable diseases, height over 170 cm, maximum age of 24, no addiction, and no history of misconduct. The associate degree in emergency medicine has 68 academic units and usually takes 2 years. In the bachelor's degree program, students must pass 130 academic units including theoretical courses, practical courses, and internships, which usually take 4 years. The faculty members of the emergency medicine group include individuals from nursing, emergency medicine, emergency medicine specialists, and anesthesiologists departments. The education of emergency medicine students is carried out theoretically and practically in educational classes and clinical skill halls of the faculty as well as in real pre-hospital and hospital environments. The activity of the Emergency Medical Services (EMS) system in Iran is independent of other emergency and security systems (Red Crescent, Fire Department, and Police). This system has a unique number, 115, for public access and assistance 24/7. Recent government policies aim to expand ambulance bases in cities, intercity roads, and main rural roads.

According to the latest guidelines from the Ministry of Health in Iran, cities with a population of 50,000 must have at least one base for deploying personnel and ambulances. On the other hand, for every additional 60,000 people above the base population, an extra ambulance station is needed. According to these instructions, there should be an ambulance station every 30 kilometers along the main roads that connect cities. The EMS providers in Iran consist of a combination of nurses and EMTs (Emergency Medical Technicians). The system's missions are carried out by two EMS providers. The personnel sent to the scene of the accident, along with the consulting physician in the dispatch unit, are responsible for providing medical services to patients and transporting them to the hospital.

### Participants

The participants included operational EMS providers who worked in both urban and rural areas in the emergency medical service of Hamadan, Iran. In addition, they had at least one year of work experience in the field of pre-hospital emergency care and held academic degrees in emergency medical technician or nursing. Working in the medical emergency system is always challenging for various reasons. Thus, it can be argued that all emergency medical personnel face professional challenges in this field. Permission was obtained from the managers of the emergency medical system to conduct the research and select the participants. The study's purpose was explained to the emergency medical personnel, and informed written consent was obtained from the EMS providers. The first person selected for an interview had 15 years of work experience. Subsequently, individuals from urban and road bases with varying work experience, degree, and ages were also included in the study. In this way, we attempted to select a sample with the maximum diversity. In this study, a total of 18 participants were included, with 11 from urban bases and 7 from rural bases. In the present study, data saturation was achieved after interviewing 14 participants. In total, eighteen male EMS providers were selected using the purposive sampling method.

### Data collection

In this study, semi-structured and face-to-face interviews were conducted to collect the necessary data. Interviews were conducted outside of the work shift and with prior coordination of the participants in the meeting room of the Central Building of the Emergency Medical System. Interviews were conducted by the Corresponding author and lasted between 45 and 60 minutes. The interviews continued until data saturation was reached. All interviews were conducted in Persian. The first question of the interview was an open-ended one: “Could you please talk about your occupational experiences?” Then, based on participants' responses, the interviews continued with more detailed questions such as "Have you encountered any challenges in your missions?" and "Are there any challenges at work or in relation to your colleagues?" Can you explain your feelings in those situations?" However, open-ended questions may not always elicit all the information needed to answer the research questions. Therefore, follow-up or guiding questions may be necessary to clarify or expand on the participant's response. These guiding questions aim to provide more context and detail to the participant's initial response, allowing the researcher to better understand the participant's perspective and experiences. Examples of guiding questions include "Can you explain more?" or "What do you mean by this?"

### Data analysis

The data were analyzed according to the Graneheim and Lundman five step method including 1. Transcribing the interviews and studying them repeatedly to obtain a general concept from them 2. Dividing the text into meaningful units 3. Summarizing meaningful units and extracting codes 4. Placing codes in subcategories and categories based on their similarities and differences 5. Organizing and extracting themes from the hidden content of interview text [15]. First, the interviews were coded after conducting the interviews and transcribing them into a Word file. The created codes were then placed in the appropriate classes. During the process of collecting information, these classes expanded, and some classes were added, removed, or merged. Then, the researcher attempted to obtain a comprehensive understanding of the subject by connecting these classes together. Finally, the main category was determined by considering the number of words, phrases, themes, or concepts present in each category. The collected data were analyzed in MAXQDA v. 2010.

#### Rigor

The criteria used to assess the accuracy of Lincoln and Goba’s research were validity, validity, reliability and transferability. The researchers thoroughly and extensively analyzed the data to ensure its credibility. Further, they employed the member check review method, in which they provided the participants with the text of the interviews and codes after extracting the initial code from the interviews. This allowed the participants to modify or confirm the codes. Furthermore, to ensure confirmability in interpreting the data, the peer review method was employed. Two experts in the field of qualitative research coded a number of unnamed interviews. Regarding dependability, the research team was divided into two groups, and each group performed the coding process individually. Then, they reached a consensus during a meeting after presenting the codes. To ensure transferability, participants of different ages, different educational degree and with different work experience took part in the study.

#### Ethical considerations

The project was approved by the Ethics Committee of Hamadan University of Medical Sciences (Code: IR.UMSHA.REC.1396.870). First, the purpose of the interview was verbally explained, and then informed consent was obtained to allow the recording of the interviews and the use of the information while ensuring its confidentiality. The participants were assured that their anonymity would be preserved, and they could withdraw at any stage of the survey.

## Results

All participants were male. Thirteen participants were married and eleven were working in urban EMS stations .The other demographic characteristics of the participants are shown in Table 1. Analyses of the data obtained from the interviews resulted in finding one theme and five categories (Table 2).

**Table 1:** Characteristics of the participants

**Characteristics of Participants**	**N (%) or Range (Median)**
Educational degree	
EMT	3(17)
BS in EMS	7(39)
BS in Nursing	5(28)
MSc in Nursing	3(16)
Years in Practice	3 – 25(13)
Age	25 – 51(36)
Gender	
Male	18(100)
Marital Status	
Married	13(72)
Single	5(28)
Ambulance station location	
Urban	11(61)
Rural	7(39)

**Table 2:** Themes and categories extracted from content analysis

**Theme**	**categories**	**Subcategories**
professional challenges	Ineffective policies	Ambiguity in pre-hospital guidelines
		inappropriate executive considerations
		inadequate staffing preparation
	multicultural and multidisciplinary factors	Incompatibility with colleagues
		difficulties with the non-homogeneous patient
		inconsiderate behavior toward patients/family
	Ambulance dispatch route problems	Ambulance crashes
		traffic congestion
		weather conditions
	legal issues	complaints against technicians
		lack of legal knowledge
	abuse against emergency medical services	Misuse of the Emergency Medical Services by Individuals
		interference of the people and patient’s family in Relief process

### Ineffective policies

EMS providers expressed concerns about the functions, practices, and policies of organizational leaders and officials, which posed a professional challenge. The main categories of Ineffective policies included "ambiguity in pre-hospital guidelines," "inappropriate executive considerations," and "inadequate staffing preparation."

### Multicultural and multidisciplinary factors

EMS providers highlighted factors related to interpersonal behaviors and interactions with colleagues, as well as hospital staff, especially with the emergency department of the hospitals and care and treatment in the pre-hospital field as professional challenges. The main category of multicultural and multidisciplinary factors was formed from three subcategories including “Incompatibility with colleagues", "difficulties with the non-homogeneous patient and “inconsiderate behavior toward patients/family.

### Ambulance dispatch route problems

Ambulance dispatch route problems refer to any difficulties or challenges that may arise during the process of dispatching an ambulance to an emergency situation. The main category of Ambulance dispatch route issues was formed from three categories, including Ambulance crashes, traffic congestion, and weather conditions.

### Legal issues

This concept emerged from participants' perceptions of numerous problems with legal and regulatory issues and complaints against technicians related to prehospital emergency work and the prosecution of this matter by judicial organizations. Most EMS providers pointed out these issues in their interviews, noting that complaints against technicians have become a routine issue.

### Abuse against the emergency medical services

This concept was derived from the EMS providers' perceptions of patient, family, and community behavior in the prehospital field. Participants reported misunderstandings, inappropriate behavior, and misconceptions of people during the interviews. The main category of abuse against the emergency medical services was formed from two categories, namely misuse of emergency medical services by individuals and interference of the people and patient’s family in relief process.

## Discussion

This study aimed to explore perceived professional challenges among EMS providers. The professional challenges faced by EMS providers are numerous and complex. Findings revealed that the main perceived professional challenges among EMS providers were Ineffective policies, multicultural and multidisciplinary factors, ambulance dispatch route problems, legal issues, and abuse against emergency medical services. These findings are discussed in what follows.

Applying and effectively implementing the guidelines can improve patient outcomes [16]. Despite the instructions and protocols, there is still a gap between the established guidelines and clinical practice [17, 18]. The present study showed that EMS providers use established protocols at the scene in a limited number of missions. Moreover, despite the improvements in providing and developing care and treatment protocols related to pre-hospital missions, emergency medical personnel often consult with a physician at a communication center to manage such missions. However, the physician's orders may not always align with the best interest of the patient or may result in unnecessary and futile interventions by technicians. This can be attributed to their lack of confidence in assessing and obtaining accurate information from patients, lack of accountability for specific cases, and fear of legal consequences. For the same reason, the participants said that they avoided consulting in most of the missions, and admitted that the lack of consultation with the physician harmed some patients in some missions. When EMS personnel do not follow the protocols and instructions, emergency patients in this field may not receive proper care [19].

One of the challenges of EMS providers in the work environment is the decisions of organizational managers regarding performing some tasks outside of duty such as deploying ambulances in some ceremonies, sports competitions or some gatherings. If an accident occurs in the area of the relevant base while deploying an ambulance, due to the dispatch of an ambulance from neighboring and farther bases from the scene of the accident, rescue will be delayed and perhaps the patient's injuries will increase. The lack of support of organizational managers for the main goals and mission of the emergency medical system and also the lack of specific guidelines and protocols for prioritizing ambulance dispatches can be involved in this matter. Sandeman and Nordmark highlighted the issue of dispatching ambulances for non-emergency missions due to the absence of specific protocols for prioritizing within the emergency medical system [3]. Access to health care and fair distribution of health services should not be hindered by factors such as age, sex, financial and economic status of the patient, or anything else [20]. In the present study, equipped ambulances are located in the areas of the city where people have a higher economic status. Thus, people receive better facilities and services compared to those residing in other areas.

The participants who took part in this study said that graduates in the EMT were not competent and skilled enough, which led to problems and outcomes such as more harm to the patients and a lot of stress for their co-workers. Afshari et al., in their qualitative study, evaluated the educational challenges faced by medical emergency students. This study identified three main categories of reasons for the graduates' lack of competence in medical emergencies in Iran: inadequate teaching methods, discrepancy between theory and practice, and curriculum reform in medical emergencies [21]. Therefore, it is recommended that medical emergency training programs in Iran should incorporate more practical, simulation, and realistic scenarios to bridge the gap between theory and practice and enhance the competence of graduates. Blackwell and Brown, in their studies on the educational issues of medical emergencies, recommended that these students, as well as other medical emergency staff, should receive training in realistic settings to enhance their skills and abilities [22, 23].

The proper relationship between healthcare workers in the workplace is essential for improving patient outcomes, staff productivity, and safety culture [24], This is particularly important in the pre-hospital field where there is a low number of personnel per mission, often with only two technicians present at the scene. Working in the pre-hospital emergency field requires collaboration, overlap, and assistance among technicians [25]. When technicians disagree on how to treat and manage patients, and these issues are influenced by their personal experiences and feelings, they face professional challenges [26]. In addition, communication with the staff of other units in the healthcare system is another issue that is addressed in terms of professional communication, which can lead to some challenges. The personnel from different units may encounter conflicts when making decisions regarding patients dispatched by the medical emergency system [4]. In the present study, participants also mentioned these issues.

In the Iranian medical emergency system and dispatch unit, only male personnel are currently active. The Iranian people have religious and cultural beliefs related to Islam [23]. These beliefs, attitudes, and perceptions of Muslim women have a direct impact on their access to health and medical services. Therefore, most families prefer that their patients be cared for by nurses and technicians of the same sex [27, 28]. The participants in this study talked about this issue as a professional challenge in dealing with these missions while not providing proper care to female patients. Access to emergency medical services is a basic right that every person should have access to regardless of gender, race and religion. However, in some Islamic societies, women face significant challenges in accessing emergency medical services, especially in situations where only men make up emergency medical personnel. Women's access to emergency medical services in Islamic societies where only men make up emergency medical personnel is a complex issue that requires a multifaceted approach. By addressing cultural, religious, and educational issues, we can work to ensure that all women have equal access to emergency medical services, regardless of gender or any other factor. In recent years, some universities of medical sciences in Iran have started recruiting female students to take training courses in the field of emergency medicine. Currently, female technicians graduated from universities are working and providing rescue in ambulance stations in limited areas of some cities.

In Iran, each shift in the emergency medical system lasts for 24 hours. Some studies have indicated that emergency medical personnel experience higher levels of fatigue and stress compared to firefighters. This is attributed to the higher number of missions they undertake in their field [29]. Awareness, concentration, judgment, mood, and performance of emergency medical personnel are significantly affected by fatigue [30]. Furthermore, fatigue can lead to memory impairment, lack of concentration in problem-solving, and decision-making, which can ultimately reduce the efficiency and effectiveness of patient care [31]. The participants in the present study reported that they were not adequately prepared to handle the demands of future missions during shifts with a high number of missions and fatigue. As a result, they were unable to provide proper care to the patients while carrying out their missions.

Richard et al. indicated that ambulance accidents among EMS providers were about 20 times more frequent than those among other staff members [32]. Alexander evaluated the impact of ambulance crashes on the mental health of technicians and found that the technicians would experience long-term psychological problems as a result of being involved in accidents and causing harm to others [33]. The results of the Brent and Beland study showed that traffic causes delays in the arrival of ambulances and fire trucks to the scene of an accident and this will increase the response time. They also showed that traffic congestion increases response times by 3.4% for EMS and 4.7% for fire [34]. Studies have shown that weather conditions such as icy or wet road surfaces and foggy air can cause problems on the road by reducing visibility and vehicle control. Also, in inclement weather conditions, emergency medical services may have more demand [35]. The results obtained in the aforementioned studies are consistent with the findings conducted in this study.

Inevitably, the occurrence of grievances among emergency medical personnel is recognized as a prevalent concern within this profession. In this study, the main sources of complaints about the care providers were the patients (53%), the medical staff (19%), and the patient's family (12%). The complaints were related to the lack of skills among the staff (20%), problems in the transmission pathway (18%), and the loss of patient's equipment (13%) [36]. Technicians in this study expressed concerns about anxiety and exhibited cautious behavior during most missions to avoid any potential complaints.

Dejean et al. reported that several factors contribute to the inappropriate use and misuse of the emergency medical system. These factors include people's lack of awareness about the medical emergency system, limited transportation options, the patient's inability to walk, and the absence of efforts to pursue conventional treatments [37]. Furthermore, Pekanoja et al. concluded that 38% of the missions did not need to transfer to the hospital, and 80% of the missions only required a visit from a general physician [38]. Additionally, Patel et al. evaluated trauma delays in road traffic injury patients and identified several contributing factors, including lack of public education, road traffic congestion, shortage of personnel and ambulances, and inappropriate location of stations [39]. Pourshaikhian et al described violence against personnel as linguistic threats, physical conflicts, insults and flout, and similar cases and emphasized that these behaviors by the people and patient’s family or people on the scene could interfere with the rescue [40]. In the present study, participants reported facing non-emergency situations, misuse of emergency medical services by people, and delays in rescue due to inappropriate interference by individuals at the scene.

## Conclusion

The results of the present study have shown that a wide range of interpersonal, social, and organizational factors contribute to professional challenges among EMS providers. While further research is needed to better understand the occupational and professional challenges in the pre-hospital field, emergency medical system managers can use the results of this study to develop effective programs to address workplace problems and challenges. These programs may include addressing staff issues at emergency medical bases, providing periodic training for new personnel, introducing the emergency medical system to the public through media, providing legal training and counseling to EMS providers, and modifying certain organizational guidelines.

## Data Availability

The datasets used and/or analysed during the current study are available from the corresponding author on reasonable request.

## Abbreviations

EMS: Emergency medical services
BS: Bachelor of Science
MSc: Masters of Sciences
EMT: Emergency Medical Technician

## References

[1] Becker TK, Gausche-Hill M, Aswegan AL, Baker EF, Bookman KJ, Bradley RN (2013). Ethical challenges in Emergency Medical Services: controversies and recommendations. Prehosp Disaster Med.

[2] French E, Casali GL (2008). Ethics in emergency medical services–Who cares? An exploratory analysis from Australia.

[3] Sandman L, Nordmark A (2006). Ethical conflicts in prehospital emergency care. Nurs Ethics.

[4] Ripley E, Ramsey C, Prorock-Ernest A, Foco R, Luckett S, Ornato JP (2012). EMS providers and exception from informed consent research: benefits, ethics, and community consultation. Prehosp Emerg Care.

[5] Bremer A, Herrera MJ, Axelsson C, Martí DB, Sandman L, Casali GL (2015). Ethical values in emergency medical services: a pilot study. Nurs Ethics.

[6] Afshari A, Torabi M, Navkhasi S, Aslani M, Khazaei A (2023). Navigating into the unknown: exploring the experience of exposure to prehospital emergency stressors: a sequential explanatory mixed-methods. BMC Emerg Med.

[7] Mirzaei A, Mozaffari N, Soola AH (2022). Occupational stress and its relationship with spiritual coping among emergency department nurses and emergency medical services staff. Int Emerg Nurs.

[8] Afshari A, Khalili A, Dehghani M, Beiramijam M, Lotf M, Noodeh F (2017). Comparing the frequency of occupational injuries among medical emergency staff and nurses of intensive care units in Hamadan. Ann Trop Med Public Health..

[9] Afshari A, Borzou SR, Shamsaei F, Mohammadi E, Tapak L (2021). Perceived occupational stressors among emergency medical service providers: a qualitative study. BMC Emerg Med.

[10] Afshari A, Borzou SR, Shamsaei F, Mohammadi E, Tapak L (2021). Emergency medical service providers’ perception of health-threatening stressors in emergency missions: a qualitative study. Ethiop J Health Sci..

[11] Torabi M, Borhani F, Abbaszadeh A, Atashzadeh-Shoorideh F (2020). Barriers to ethical decision-making for pre-hospital care professionals. Nurs Ethics.

[12] Lewis S (2015). Qualitative inquiry and research design: Choosing among five approaches. Health Promot Pract.

[13] Hsieh H-F, Shannon SE (2005). Three approaches to qualitative content analysis. Qual Health Res.

[14] Tong A, Sainsbury P, Craig J (2007). Consolidated criteria for reporting qualitative research (COREQ): a 32-item checklist for interviews and focus groups. Int J Qual Health Care.

[15] Graneheim UH, Lundman B (2004). Qualitative content analysis in nursing research: concepts, procedures and measures to achieve trustworthiness. Nurs Educ Today.

[16] Lugtenberg M, Burgers J, Westert G (2009). Effects of evidence-based clinical practice guidelines on quality of care: a systematic review. BMJ Qual Saf.

[17] Grol R, Grimshaw J (2003). From best evidence to best practice: effective implementation of change in patients' care. Lancet.

[18] Van Achterberg T, Schoonhoven L, Grol R (2008). Nursing implementation science: how evidence-based nursing requires evidence-based implementation. J Nurs Scholarsh.

[19] Ebben RH, Vloet LC, Verhofstad MH, Meijer S, Mintjes-de Groot JA, van Achterberg T (2013). Adherence to guidelines and protocols in the prehospital and emergency care setting: a systematic review. Scand J Trauma Resusc Emerg Med.

[20] Symons P, Shuster M (2004). International EMS Systems: Canada. Resuscitation.

[21] Afshari A, Khodaveisi M, Sadeghian E (2021). Exploring the educational challenges in emergency medical students: a qualitative study. J Adv Med Educ Prof.

[22] Blackwell TH, Halsey RM, Reinovsky JH (2016). Emergency medical technician training for medical students: a two-year experience. Prehosp Emerg Care.

[23] Brown D, Zimitat C (2012). On the road: medical students’ experiences on paramedic placements. Med Teach.

[24] Almost J, Wolff AC, Stewart-Pyne A, McCormick LG, Strachan D, D'souza C (2016). Managing and mitigating conflict in healthcare teams: an integrative review. J Adv Nurs.

[25] Larkin GL, Fowler RL (2002). Essential ethics for EMS: cardinal virtues and core principles. Emerg Med Clin North Am.

[26] Gunnarsson B-M, Stomberg MW (2009). Factors influencing decision making among ambulance nurses in emergency care situations. IntEmerg Nurs.

[27] Borhani F, Abbaszadeh A, Moosavi S (2014). Status of human dignity of adult patients admitted to hospitals of Tehran. Journal of medical ethics and history of medicine..

[28] Shahriari M, Mohammadi E, Fooladi MM, Abbaszadeh A, Bahrami M (2016). Proposing codes of ethics for Iranian nurses: a mixed methods study. J Mixed Methods Res.

[29] Takeyama H, Itani T, Tachi N, Sakamura O, Murata K, Inoue T (2009). Effects of a modified ambulance night shift system on fatigue and physiological function among ambulance paramedics. J Occup Health.

[30] Dawson D, Zee P (2005). Work hours and reducing fatigue-related risk: good research vs good policy. JAMA.

[31] Jansen N, Van Amelsvoort L, Kristensen T, Van den Brandt P, Kant I (2003). Work schedules and fatigue: a prospective cohort study. Occup Environ Med.

[32] Reichard AA, Marsh SM, Tonozzi TR, Konda S, Gormley MA (2017). Occupational injuries and exposures among emergency medical services workers. Prehosp Emerg Care.

[33] Alexander DA, Klein S (2001). Ambulance personnel and critical incidents: impact of accident and emergency work on mental health and emotional well-being. Br J Psychiatry.

[34] Brent D, Beland L-P (2020). Traffic congestion, transportation policies, and the performance of first responders. J Environ Econ Manag.

[35] Rhodes N, Pivik K (2011). Age and gender differences in risky driving: The roles of positive affect and risk perception. Accid Anal Prev.

[36] Colwell CB, Pons PT, Pi R (2003). Complaints against an EMS system. J Emerg Med.

[37] Dejean D, Giacomini M, Welsford M, Schwartz L, Decicca P (2016). Inappropriate ambulance use: a qualitative study of paramedics' views. Healthcare Policy.

[38] Pekanoja S, Hoikka M, Kyngäs H, Elo S (2018). Non-transport emergency medical service missions–a retrospective study based on medical charts. Acta Anaesthesiologica Scandinavica.

[39] Patel A, Vissoci JRN, Hocker M, Molina E, Gil NM, Staton C (2017). Qualitative evaluation of trauma delays in road traffic injury patients in Maringá, Brazil. BMC Health Serv Res.

[40] Pourshaikhian M, Gorji HA, Aryankhesal A, Khorasani-Zavareh D, Barati A (2016). A systematic literature review: workplace violence against emergency medical services personnel. Arch Trauma Res..

